# Multilingualism and augmentative and alternative communication in South Africa – Exploring the views of persons with complex communication needs

**DOI:** 10.4102/ajod.v8i0.507

**Published:** 2019-04-24

**Authors:** Kerstin M. Tönsing, Karin van Niekerk, Georg Schlünz, Ilana Wilken

**Affiliations:** 1 Centre for Augmentative and Alternative Communication, University of Pretoria, Pretoria, South Africa; 2 Human Language Technology Research Group, Meraka Institute, Council for Scientific and Industrial Research, Pretoria, South Africa

## Abstract

**Background:**

Augmentative and alternative communication (AAC) can assist persons with complex communication needs to communicate competently with a variety of communication partners in a variety of contexts. However, AAC systems and intervention often do not take multilingual aspects into consideration.

**Objective:**

This small-scale exploratory study had three aims, namely: (1) to describe the self-reported language skills of multilingual South African adults using AAC, (2) to describe the languages and communication modalities they used in interaction and (3) to obtain their views regarding access to various languages.

**Methods:**

Twenty-seven adults using AAC were recruited via an empowerment programme, as well as an email list for persons interested in AAC, and provided responses to a questionnaire. To compensate for access and written language challenges, the questionnaire was administered with help and/or as a face-to-face interview where needed. Responses were analysed using mostly descriptive statistics.

**Results:**

Participants generally could not express themselves in all the languages they understood and were regularly exposed to. Speech-generating devices specifically gave access almost exclusively to English. Participants expressed a desire to increase their expressive language repertoire, and mentioned both limitations of communication technology as well as their own literacy skills as barriers to overcome in this regard.

**Conclusion:**

In order for multilingual South African adults using AAC to express themselves in multiple languages, appropriate AAC systems and interventions as well as literacy learning opportunities need to be developed and provided.

**Keywords:**

adult, augmentative and alternative communication; multilingualism; complex communication needs, language and communication skills; self-report, views.

## Introduction

Augmentative and alternative communication (AAC) encompasses techniques, symbols, strategies and aids that can be used by persons with complex communication needs whose speech is too limited to meet all their communication needs (American Speech-Language-Hearing Association 2018). Augmentative and alternative communication systems include aided systems (e.g. speech-generating devices [SGDs] and communication boards) and unaided options (e.g. gestures and manual signs from a sign language). The focus of this article is on persons who need AAC long term for primarily expressive purposes (i.e. their ability to comprehend spoken language is relatively intact). For this group, a linguistic method of expression that allows the generation of self-composed novel utterances is typically desirable, as this allows true autonomy in communication (Light & McNaughton 2012). A method of expression that additionally aligns to the language(s) used within the communities they are part of allows for community integration and direct communication access to frequent communication partners without the need for translation.

Orthography-based AAC methods allow for the composition of novel messages, but persons using them need to be literate in the appropriate natural languages. Partners also need to be literate, unless text-to-speech (TTS) synthesis^1^ is used via SGDs. In the absence of literacy skills, picture symbol-based AAC systems may be used, whereby vocabulary items in one or more languages are represented by picture symbols or picture symbol sequences, and made available on communication boards or SGDs. These pictures are typically custom-designed collections that are integrated into the SGD software and/or are commercially available. Examples include Picture Communication Symbols^TM^ ^2^, SymboStix^3^, Widgit^4^ and Minspeak^®^ symbols. Only systems that include a large, relevant vocabulary composed of a variety of word types can presume to give access to a degree of novel utterance generation (Light & McNaughton 2012).

There is as yet a dearth of research in AAC implementation for multilingual clients (Kulkarni & Parmar 2017; Soto & Yu 2014). In spite of the prevalence of multilingualism amongst the general population as well as persons with communication disorders, studies in communication development, disorders and AAC have primarily focussed on monolingual populations and monolingual interventions (Kohnert 2013). Studies that document the implementation of bi- or multilingual AAC systems seem at present to be limited to case studies and anecdotal reports (e.g. Harrison-Harris 2007; Stewart 2017). There may be a number of reasons. There is a prevailing notion that multilingualism may be difficult for clients with communication disorders, especially those who experience these disorders from a young age (De Valenzuela et al. 2016; Drysdale, Van der Meer & Kagohara 2015; Gutierrez-Clellen 1999; Levey & Sola 2013; Yu 2013). As yet, no empirical evidence supports these suppositions (Kay-Raining Bird, Genesee & Verhoeven 2016; Kohnert 2013; Kohnert & Medina 2009). However, communication interventionists may still advise clients and families to use only one language (Yu 2013). Much of the research and technology developments in AAC have been conducted in high-income countries, and specifically in the USA. Multilingualism has been less prevalent in the USA than in other countries (Grosjean 2013), with English clearly established as the majority language (Kaplan 2015). However, an increase especially in the Hispanic population in the USA has led to renewed interests in the integration of various languages in the US education system, and has also increased the recognition of the benefits and value of multilingualism (Lee & Wright 2014; Lozanso-Alonso 2017). Even so, developments in AAC have historically focussed primarily on persons from monolingual and specifically English language backgrounds (Bridges 2004). Parents of children in need of AAC who come from non-English backgrounds have remarked on challenges in AAC service delivery related to multilingual aspects, including a lack of AAC systems that give access to languages other than English (Huer, Parette & Saenz 2001; Pickl 2011; Singh et al. 2017).

The act of communication and the use of language to achieve this is not only an act of sharing information, but at the same time an act to, consciously or unconsciously, assume, assert and recreate one’s identity, power status and group affiliation (Bordieu 1991; Norton & Columbia 2011). South African adolescents, for example, described their home language as their ‘structure’, their ‘skeleton’ and ‘part of who you are’ (Ndlangamandla 2010:67), and as ‘my being and my life’, ‘my culture’ and ‘my grounding’ (Bristowe, Oostendorp & Anthonissen 2014:232–233). For multilingual speakers, choice of language and use of mechanisms such as code switching and code mixing may not be neutral acts, but acts of identity that may also serve to show respect, promote group cohesion or align or distance oneself from communication partners (Bristowe et al. 2014; McKinney 2013; Ndlangamandla 2010). McKinney (2013:25) described, for example, how one of the participants in her study (an adolescent South African girl) viewed her ability to use different languages, including non-standard varieties, as an ability to ‘perform different identities’. By being able to fluently speak isiXhosa, English and what is termed ‘Tsotsitaal’, she could integrate into various communities seamlessly. Achieving social closeness with others through communication may also be contingent upon being able to express oneself in a given language, such as one’s home language when speaking to a close family member (Tönsing et al. 2018).

As language is a tool of power, access to and choice of one’s use of languages is integral to human freedom and assumes the status of a democratic right (May 2001). The South African Constitution (1996, Section 30) grants every citizen the right to use the language of his or her choice. As persons who rely on AAC are already at risk of powerlessness because of their use of less conventional methods of communication, limiting access to different natural languages may be a further act of exclusion.

A person and community’s use of and competence in multiple languages is influenced by a variety of factors, including top-down factors such as language policy in education, and bottom-up factors related to the views, opinions and choices of individuals and communities regarding the use of different languages (Webb 2010). In South Africa, English (the first language of only 9.6% of the population) dominates the education and business spheres (Kathard et al. 2011; Khokhlova 2015; Webb, Lafon & Pare 2010), in spite of the official equal status of all 11 official languages granted by the South African Constitution post-apartheid (The *Constitution of the Republic of South Africa*, Act 108 of 1996, Section 6[1]). The majority of learners in the basic education system, for example, are educated in English, despite the fact that this is not their home language (Department of Basic Education 2011). While some authors argue that this is resulting in language shift and some cases, language loss (De Klerk 2002a; Kamwangamalu 2003), others maintain that there is little evidence of widespread and extensive language shift towards English and language loss of the other 10 official South African languages (Bristowe et al. 2014, Coetzee-Van Rooy 2012, Ndlangamandla 2010). These authors report that many South Africans seem to be increasingly multilingual, with the home language (defined as the language used most frequently in the home, cf. Coetzee-Van Rooy 2012) being maintained for use in the home, English being added as the language of education and the media, and other languages being added to enhance community integration or work opportunities (Coetzee-Van Rooy 2012). In multiple surveys amongst high school and university students from African and Afrikaans language backgrounds, Coetzee-Van Rooy (2016) found that students regarded the ability to use more than one language as very important for South Africans, and suggested communication access, respect for diversity and social cohesion as reasons for learning more than one language.

The question arises as to how multilingual South Africans who use AAC negotiate the multilingual South African context. Their ability to express themselves in multiple languages not only relies on opportunities to learn different languages and their interest in doing so, but also on appropriate AAC intervention, including access to relevant AAC systems. As communication intervention in South Africa (including AAC intervention) is primarily provided in English (Dada, Murphy & Tönsing 2017; Kathard et al. 2011), and commercially available AAC systems are predominantly available in English (Bridges 2004), it seems probable that these factors would restrict access to expression in other languages. Understanding to what extent South African adults using AAC have the desire and ability to use multiple languages can assist in directing the development of appropriate intervention services as well as appropriate AAC systems.

### Aims

This exploratory study had three aims: (1) to describe the self-reported language skills of multilingual South African adults using AAC, (2) to describe their use of different languages and modalities in interactions and (3) to obtain their views regarding their desire to use multiple languages.

## Research method and design

### Design

A quantitative descriptive survey was conducted using a questionnaire. This design was considered appropriate for the current project because it allowed the researchers to reach a larger sample of participants and also provided a response format (primarily closed-ended questions) that did not require participants to provide extensive narrative responses – a process that can be physically fatiguing for persons with physical disabilities. The researchers used various forms of recruitment and various formats of the questionnaire (hard copy, emailed and online) to include more participants. This meant that nine participants completed the questionnaire without the presence of trained research assistants, and the degree to which the responses received truly reflected their own opinions could not be monitored directly.

### Participants

Participants had to (1) be 18 years or older, (2) have a level of autonomy in expression that ensured that their thoughts and opinions could be captured in an unbiased way and (3) use a form of AAC because of having complex communication needs. Participants were recruited via an empowerment programme for young adults using AAC, and also via an email list of persons interested in AAC. This empowerment programme for young adults using AAC is run annually at a university-based institution. Participants take part in communication and empowerment training activities for the duration of 1 week. Participants were approached in person during programme weeks, and alumni were approached via email or text message. A total of 24 persons were approached, and 21 gave consent to participate. As these participants were all known to the researchers in person, the researchers were able to verify that the participants met all the selection criteria. A further seven persons responded to an invitation sent to a South African email list for persons interested in AAC (including persons who use AAC, family members and service providers) administrated by a university-based institution. This list contains 1138 email addresses; however, because many email addresses may not belong to multilingual South African adults who use AAC, it is not possible to report a response rate. To ensure that participants met the selection criteria, they were asked to confirm this by answering three questions related to the three criteria. Of the seven respondents, one indicated that he did not require AAC because his speech was functional, and this respondent was therefore excluded from the study. Of the remaining six respondents to the emailed invitation, only one was not directly or indirectly (via service providers) known to the researchers. However, the respondent contacted the researchers via email after completing the study, and through the emailed exchanges seems very likely that he met the selection criteria. Therefore, the researchers are confident that all respondents met the selection criteria.

The recruitment and the fact that the questionnaire was only provided in English may have biased the sample towards those with English skills, literacy skills, higher levels of education and a level of professional support. For example, only persons who were in some direct or indirect way linked to the university-based institution (e.g. via their service provider or a family member) had a chance to be included in the study. The empowerment programme at the university is offered primarily in English, although participants may attend if their personal assistants can act as translators. Also, participants in this programme are required to have a means of expressing their own thoughts, and this indirectly implies that many have some measure of literacy skills. Two respondents (both participants of the empowerment programme) who rated their understanding of English as *a little* also participated in the survey. For both respondents, the personal assistants or research assistants helping them to complete the survey were fluent in both English and the home language (Afrikaans and Setswana respectively) and translated the questions for the participant. All respondents to the emailed invitation had good literacy and good English skills.

Most participants were men and between the ages of 18 and 29 years (*M* = 30.1, *SD* = 13.3, range = 18–73). They resided in seven of the nine South African provinces, with the biggest group (*n* = 9) residing in Gauteng. They came from a variety of language backgrounds, with 18 participants (67%) coming from African language backgrounds, while nine (33%) came from English or Afrikaans language backgrounds. While it is clear that there is oversampling of persons from English- or Afrikaans-speaking backgrounds (who make up 23% of the general population according to Statistics South Africa 2012), and that such a small sample cannot be representative, it should still be noted that 8 of the 11 official languages were included as home languages. Also, the five home languages represented most frequently amongst the participants (see Table 1) constitute five of the six most frequently spoken home languages in the general population (Statistics South Africa 2012).

**TABLE 1:** Participant demographics.

Variable		Frequency (*N*)	Percentage (%)
Sex	Male	17	63.0
	Female	10	37.0
Age	18–24 years	12	44.4
	25–29 years	4	14.8
	30–34 years	6	22.2
	35 years and older	5	18.5
Living arrangements†	With parents or family	22	81.5
	In a residence	9	33.3
	Independent or paid carer	1	3.7
	With partner	2	7.4
Urban or rural‡	Urban	20	74.1
	Peri-urban	4	14.8
	Rural	4	14.8
Highest level of formal education	None	3	11.1
	Secondary school	16	59.2
	Post-school education	8	29.6
Onset of speech difficulties	Birth	16	59.3
	Childhood	5	18.5
	Teenager	2	7.4
	Adult	4	14.8
Diagnosis	Cerebral palsy	15	55.6
	Traumatic brain injury	3	11.1
	Parkinson’s disease	1	3.7
	Brain stem stroke	1	3.7
	Post-illness	3	11.1
	Motor neuron disease	1	3.7
	Friedrich’s ataxia	1	3.7
	Opercular syndrome	1	3.7
	Unknown	1	3.7
AAC intervention	Currently	5	18.5
	Previously but not currently	19	70.4
	Never	3	11.1
Home language	isiZulu	7	25.9
	Afrikaans	6	22.2
	Setswana	5	18.5
	isiXhosa	3	11.1
	English	3	11.1
	Xitsonga	1	3.7
	Tshivenda	1	3.7
	isiNdebele	1	3.7
Multilingual§	-	27	100.0
Help received to complete survey	Research assistant	17	63.0
	Other	4	14.8
	None	6	22.2

AAC, augmentative and alternative communication.

† , Some respondents marked more than one option because they spent time in different living arrangements, for example living in a residence for periods of time and living with their families the rest of the time.

‡ , One respondent spent some of his time in a rural area and the rest of his time living in an urban area.

§ , For the purpose of this study, being multilingual was defined as being able to understand at least two languages well (measured by self-report). The reason for using this definition rather than more traditional ones focussing on the use of multiple languages (cf. Grosjean 2013) is that participants may have been restricted in their ability to use multiple languages (although able to understand them) by factors such as access to appropriate AAC).

Table 2 summarises the AAC systems and strategies used by the participants which had design features to allow a level of novel utterance generation.

**TABLE 2:** Orthography- and picture-based augmentative and alternative communication systems and strategies used by the participants.

Description	Frequency (*n*)†	Percentage (%)
SGDs: Types used‡		
Dedicated	2	7.4
Non-dedicated		
Windows laptop with TTS software	10	37.0
iPad with communication app(s)	7	25.9
Android tablet with communication app	1	3.7
Android cellular phone with communication app	2	7.4
Other device functioning as SGD§	1	3.7
SGDs: Symbols used		
Orthography (alphabet)	20	74.0
Picture symbols¶	7	25.9
SGDs: Access		
Direct access without selection aid (e.g. using body part to activate a cell or touch screen)	11	40.7
Direct access with selection aid(s) (external keyboard, conventional mouse, tracker ball, headmouse, headstick, etc.)	10	51.9
Scanning using switches	2	7.4
SGDs: Type and language of speech output		
TTS only		
English	19	82.6
Afrikaans	1	4.3
English TTS and recorded speech		
isiZulu	2	8.7
Afrikaans	1	4.3
Communication board with picture symbols	12	44.4
Alphabet board	11	40.7
Typing on a cell phone (without speech generation)	20	74.1
Typing on a computer (without speech generation)	18	66.6
Writing on paper	1	3.7
Tracing letters on the floor with finger	1	3.7
Tracing letters on a person’s body with finger	1	3.7

SGD, speech-generating device; TTS, text-to-speech.

† , *n* refers to the number of participants using these methods.

‡ , a total of 21 participants used an SGD – Two participants each used two types of SGDs (one used a laptop and an iPad, and the other an iPad and an Android tablet).

§ , one participant used a talking dictionary device as an SGD.

¶ , six participants used picture symbols in addition to orthography on their SGDs. The other participant used only picture symbols on her SGD, and did not use any form of orthography-based communication. She reported no written language proficiency in any language. She had never received formal schooling.

Besides what is traditionally described as aided AAC (i.e. picture symbol-based communication boards or books, SGDs and alphabet boards), other methods such as typing on a cell phone or a computer (without voice output) were also used.

### Materials

A questionnaire was developed by the authors to address the research aims. The questionnaire consisted of 33 predominantly closed questions. Of the questions, nine pertained to demographic background information, 16 to the adults’ language and communication skills and four to the desire for increased or additional access to languages. Two questions pertained to desired communication technology features related to multilingualism – the responses to these two questions were not analysed for the purpose of this article. One open-ended question solicited any further comments that participants wanted to share while a final question asked about the assistance the participant had received in answering the questionnaire.

In the section of the questionnaire asking about participants’ desire for increased or additional access to languages, a list of possible reasons for wanting access to (a) particular language(s) was included. The reasons given were based on the previous literature that showed that social cohesion, personal identity, access to various communication partners and a desire to learn and practise using different languages were some of the main reasons that South African adolescents and adults valued multilingualism (Bristowe et al. 2014; Coetzee-Van Rooy 2016). This section also included a list of possible reasons as to why access to the identified languages was currently not as desired. The reasons given were based on the previous literature pertaining to barriers to multilingualism experienced by persons using AAC (Soto & Yu 2014, Tönsing et al. 2018). In the acknowledgement that the closed options given as reasons in these questions might not capture participants’ perspectives fully, an ‘other’ category was provided in each case, where participants could write their own reasons.

The final version of the questionnaire was available in hard copy format, electronic (Microsoft Word^TM^ and Adobe PDF^TM^) format and also as an online survey, which was developed using the Qualtrics Research Suite^5^™ survey software. These different formats also aimed to increase the range of response formats available to potential respondents.

### Procedure

Thirteen participants (all recruited via the empowerment programme) completed the survey at the university, because they attended the programme there. Of these 13, one completed the hard copy questionnaire independently. The researchers themselves or trained research assistants administered the survey to the other 12 in a face-to-face interview format. Five alumni of the programme were visited in their homes and researchers or research assistants administered the survey as a face-to-face interview. Most of these alumni had limited Internet access and therefore would have had difficulty responding via email or the online survey. A total of five research assistants were involved in data collection. All had extensive experience in working with persons using AAC, and four had a postgraduate qualification in AAC. They were all familiar with the persons whom they interviewed as they acted as facilitators during the empowerment programme. They were given an introduction to the study and the questionnaire was discussed with them. Response options such as auditory scanning were discussed and demonstrated for particular participants. The assistance given to participants entailed reading the questions and response options to the participants, and transcribing the responses the participants gave by using AAC.

Three further alumni were contacted via email and responded to the emailed invitation within 10 days, without additional reminders. The general invitation to the email list was sent out three times; each time 10 days apart. The six relevant responses to this invitation were all received within 30 days of the first email. All together, these nine respondents either completed the emailed questionnaire (which was either emailed or faxed back) or the online survey. Five of these nine respondents reported completing the survey independently, while four had help from a family member, personal assistant, friend and therapist, respectively.

### Analyses

Information from the questionnaires was captured in the statistical programme SPSS by the first author. The data were analysed using mainly descriptive statistics. Nonparametric inferential statistics (Wilcoxon signed-rank test) were also used to determine differences between skills in their home language versus English for a subgroup of participants.

### Ethical considerations

Ethics approval for the study was obtained from the authors’ respective institutions (169/2016 [CSIR] and GW20160319HS [UP]). Information letters and consent forms were provided in easy English enhanced with pictures explaining all aspects of the study. When trained research assistants were assisting potential participants, the information letter and consent form were read out to participants as needed. Participants were informed that their participation was entirely voluntary, that they could withdraw at any time. Participants recruited via the empowerment programme were also assured that their decision to take part or not take part in the study would not in any way influence their future involvement in the programme. They were informed that their participation held neither risks nor benefits for them. They could opt to receive any reports published from the project. Confidentiality is maintained as all identifying data have been removed from this article, and raw data are kept securely with access restricted to researchers and research assistants.

## Reliability and validity

Various aspects were taken into consideration during the development of the questionnaire to enhance its face and content validity. The questionnaire contained mainly closed-ended questions in order to minimise demands for writing, because composing lengthy text is physically straining for many individuals with physical disabilities. Furthermore, some questionnaires were administered with help, and closed-ended questions were deemed to reduce the risk that the participants’ answers were inadvertently interpreted or embellished by those assisting them. Questionnaires were composed in easy English. A speech-language therapist with a master’s degree in AAC and extensive experience in AAC intervention provided feedback on the first version of the questionnaire. Three additional background questions regarding current and previous communication intervention were added following her suggestions.

The first author checked all the responses to the survey for completeness and any possible inconsistencies (e.g. reporting the ability to write in a particular language but not read in it). In two cases, data were missing (once from an online questionnaire and in one case from a face-to-face administered questionnaire). In two other cases, responses needed to be clarified (once from a faxed questionnaire and once from a face-to-face administered questionnaire). The first author made contact with these four participants via email, WhatsApp or in person and obtained the missing data or clarified the responses.

To ensure that the data were captured reliably in SPSS, a research assistant checked it against all the original questionnaires. She noted any discrepancies. The first author checked the discrepancies and corrected entries where necessary. Only three discrepancies out of 3864 entries needed correction.

## Results

The results are presented according to the three aims of the study, namely: (1) to describe the self-reported language skills of multilingual South African adults using AAC, (2) to describe their use of different languages and communication modalities in interactions and (3) to obtain their views regarding a desire to use multiple languages.

### Self-reported language comprehension and written language skills

Participants were requested to rate their proficiency in spoken language comprehension, reading and writing on a scale from 1 (*not proficient at all*) to 5 (*very proficient*) in relation to the 11 official South African languages, as well as any additional languages. On average, participants understood multiple languages (*M* = 5.3, *SD* = 2.9, range = 2–11), with African language participants (*n =* 19) understanding more languages (*M* = 5.8, *SD* = 2.8, range = 2–11), than participants from English or Afrikaans home language backgrounds (*n = 8, M* = 4.2, *SD* = 3.0, range = 2–11). Participants understood an average of 2.8 languages well (rated as 4 – *understand quite a lot*, or as 5 *– understand everything*), with a standard deviation of 1.1 (range = 2–6). All rated their proficiency in their home language as high (*M* = 4.9, *SD* = 0.3, range = 4–5). All the participants understood English, with all but two rating their proficiency as 4 or 5. Two participants rated their proficiency at 2 (*understand a little*). Both of these participants had congenital disabilities and had never received any formal schooling. It is clear that the sample mirrors the multilingual South African population, and also the pervasiveness of English.

Regarding writing skills, most participants reported being able to read in multiple languages (*M* = 3.6, *SD* = 2.3, range = 1–11), and also write in multiple languages (*M* = 2.7, *SD* = 1.7, range = 0–8). The number of languages in which they were able to read well (rated as 4 or 5) was 1.7 on average (*SD =* 0.9, range = 0–4), while the number of languages in which they were able to write well (rated as 4 or 5) was 1.6 (*SD =* 0.9, range = 0–3). Regarding their home language, reading and writing proficiency was, on average, rated at 3.7 (*SD* = 1.5, range = 1–5) and 3.5 (*SD* = 1.7, range = 1–5), respectively.

#### English versus home language skills

**FIGURE 1:**
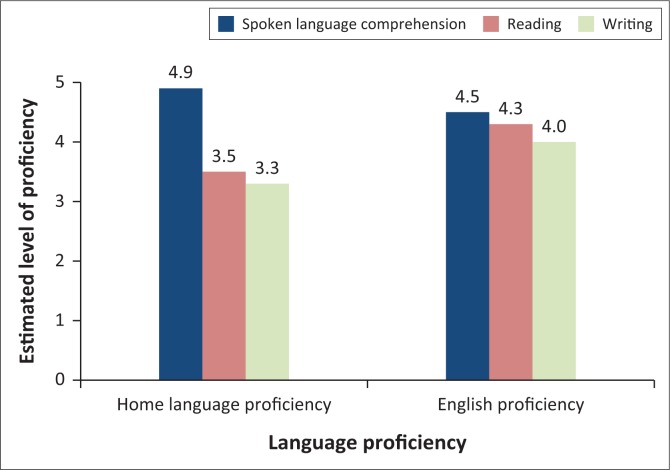
Home language versus English proficiency in spoken language comprehension, reading and writing for participants from non-English backgrounds.

The proficiency of spoken language comprehension, reading and writing in the home language was compared to that in English for participants from non-English backgrounds (*n* = 24). Results are depicted in Figure 1.

Participants’ comprehension of their home language was generally better (*M* = 4.9, *SD* = 0.2, range = 4–5) than their understanding of English (*M* = 4.5, *SD* = 0.8, range = 2–5). Conversely, written language skills were generally better in English, with reading skills estimated at an average of 4.3 (*SD* = 1.2, range = 1–5) and writing skills at 4.0 (*SD* = 1.4, range = 1–5). In the home language, reading skills were estimated at an average of 3.5 (*SD* = 1.5, range = 1–5) and writing skills at 3.0 (*SD* = 1.7, range = 1–5). A Wilcoxon signed-rank test revealed statistically significant differences between participants’ spoken language comprehension in their home language versus English on a 5% level of significance, *z = −*2.13, *p* = 0.03, with a small effect size (*r* = 0.31), and between their home language and English reading skills, *z = −*2.00, *p* = 0.045, with a small effect size (*r* = 0.28). No statistically significant difference was found between writing skills in the home language and in English, *z = −*1.84, *p* = 0.065.

### Languages and modalities used in interactions

Participants were requested to indicate which modalities, and which languages they used for expressive purposes in face-to-face interactions. They were also asked to rate the frequency with which they used the modalities and the various languages on a scale of 1 (*never*) to 5 (*very often*). Although all participants used one or more forms of non-linguistic unaided communication (such as vocalisations, gestures and eye-pointing), these forms are typically not linked to a particular spoken language and are therefore not focussed on in the results. Regarding modalities, 26 participants used orthography-based methods – mostly this entailed typing on a cell phone. Overall, these forms were rated to be used *quite often* (*M* = 4.2, *SD* = 1.4). Alphabet boards, communication boards and SGDs were used by 24 of the participants, and, on average, rated to be used *quite often* (*M* = 3.5, *SD* = 1.5), while speech was used by 19 participants (despite having complex communication needs) and rated to be used with an average frequency of 3.3 (i.e. *sometimes, SD =* 1.8).

Regarding languages used in face-to-face interactions, participants used an average of 2.1 different languages (*SD* = 0.7, range = 1–4). As expected, they used fewer languages in interaction than they understood. Of the 24 participants from non-English backgrounds, 19 used their home language in face-to-face interactions, primarily by using speech (*n* = 17), and, to a lesser extent, orthography-based methods (*n* = 10). Only six participants from this group (25%, *n* = 24) used communication boards, alphabet boards or SGDs to express themselves using their home language – four of these were from Afrikaans language backgrounds (*n* = 6) and two from African language backgrounds (*n* = 19). Within this group, 18 participants had SGDs, but only one had TTS synthesis available in her home language (using a low-quality freely available Afrikaans synthetic voice). Three other participants (one from Afrikaans and two from isiZulu language backgrounds) used recorded speech in their home language (in addition to English TTS) on their SGDs. Recorded speech does not easily allow the spontaneous generation of novel utterances, and is therefore more limiting. Five participants in this group could not use their home language in face-to-face interactions. All English participants used English through orthography-based methods and English TTS synthesis on their SGDs in face-to-face interactions.

Only three participants used SGDs to give access to more than one language – through using both recorded speech and TTS synthesis. Alphabet boards were used in two languages by five participants – interestingly always in English and Afrikaans. Four participants had either language as a home language, while for the fifth participant, neither language was her home language. None of the participants used a picture-based communication board in more than one language. Of the 19 participants who used speech, 17 spoke more than one language. Of the 26 participants who used orthography-based forms of communication, only 11 used this method in more than one language. Overall, three participants (all from African language backgrounds) used only one language to express themselves in face-to-face interactions.

As the use of different languages in face-to-face interactions is not only dependent on ability to do so, but also on partners, participants were also asked to indicate which languages others used with them in face-to-face interactions within a typical week. On average, partners used 3.6 different languages when interacting face-to-face with the participants (*SD* = 2.3, range = 1–11), as compared to the 2.1 different languages that participants typically used.

### Desire to use multiple languages

Of the participants, 23 wanted to use additional languages, or wanted to increase their use of languages they were already using in interaction. Across these participants, access to another language was mentioned 48 times overall. The language mentioned most frequently was isiZulu (mentioned 10 times), followed by Afrikaans (mentioned eight times) and Setswana (mentioned eight times). Access to English was mentioned three times – all three participants were from non-English backgrounds. Regarding access or increased access to their home language, this was desired by 19 participants – understandably all from non-English backgrounds.

**FIGURE 2:**
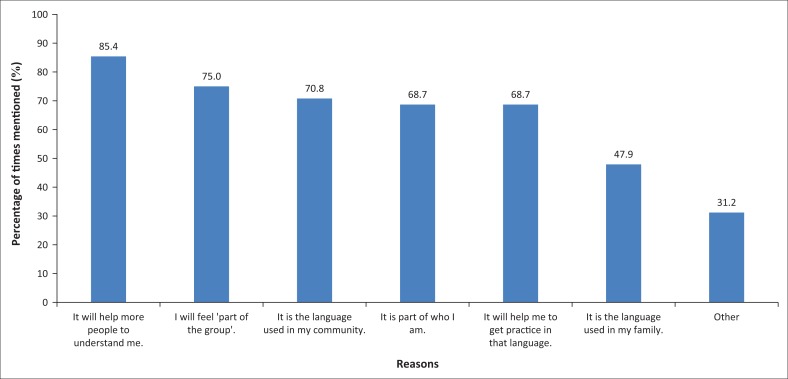
Frequency with which reasons for wanting increased or additional access to languages were chosen by the participants.

Participants were also asked to select the reasons why they required increased or additional access for each language they mentioned, by ticking appropriate options from a list of six possible reasons. The results are summarised in Figure 2.

Overall, the reason indicated most frequently was that more people would be able to understand them. Group cohesion or group identity was mentioned with the next highest frequency, followed by the fact that this was the community’s language, that it was part of their identity and that it would provide them opportunity to practise using that language. The fact that this was the language used in the family was mentioned with the lowest frequency – presumably because it would typically pertain to a limited number of languages. Participants were also able to add their own reasons in an open-ended sub-question. Additional reasons mentioned included (1) wanting to learn additional languages (mentioned six times), (2) that the particular languages would be useful for vocational and volunteer activities that participants were involved in or wanted to get involved in (mentioned five times), including acting as a chairman for a disability organisation, co-presenting workshops and preaching at church and (3) that it would facilitate understanding by specific people or groups (mentioned three times), namely personal assistants, friends and African people in general. One participant mentioned that, by being able to communicate in Afrikaans, she could ‘accommodate’ people at work whose home language was Afrikaans, even though they all understood English.

**FIGURE 3:**
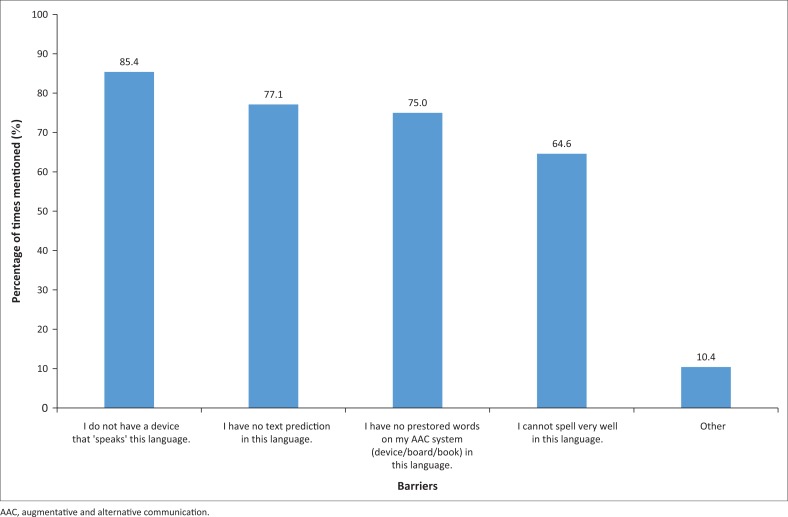
Frequency with which specific barriers prevented access to different languages.

Participants were asked to select, from a list of four options, barriers that prevented them from currently using the desired languages at all or as much as they wanted to. They could also add additional reasons on open-ended sub-questions. The results are reported in Figure 3.

Lack of access to that language through an SGD was mentioned most frequently, followed by lack of text prediction in the language, lack of pre-stored words or phrases on an AAC system in that language and inadequate spelling skills. Additional barriers mentioned included poor understanding in the desired language (mentioned four times).

Four participants did not want increased or additional access to languages. Two of these were from English-speaking backgrounds and used SGDs with English synthetic voices, as well as orthography-based methods to communicate in Afrikaans. The two other participants relied mostly on speech, but also used orthography-based methods to communicate.

## Discussion

### Outline of the results

From the data, it is clear that all participants could understand more than one language, and were exposed to multiple languages within their everyday activities. This is unsurprising given the multilingual South African context (Coetzee-Van Rooy 2012). However, their expressive abilities did not mirror the extent of multilingualism exhibited in their comprehension and context. While this may be a general trend amongst persons in multilingual contexts, participants in this study seem to have faced challenges with access to multiple languages because of limitations in the communication methods at their disposal. Particularly, more than half of the participants from non-English backgrounds (54%) did not have access to their home language via orthography-based methods of communication or picture symbol-based AAC systems. Similarly, what may be regarded as traditional forms of aided AAC (communication boards or books, alphabet boards and SGDs) mostly did not give the participants access to more than one language. Limited literacy skills in some of the languages they understood seemed to pose another barrier to their ability to express themselves in multiple languages. These results confirm that access to multiple languages for persons using AAC remains a challenge, as suggested in other qualitative investigations (Huer et al. 2001; Pickl 2011; Singh et al. 2017).

Most participants expressed a desire to use additional languages or increase their use of the languages they were already using. Similar to the respondents without disabilities in the study by Coetzee-Van Rooy (2016), adults who used AAC in the current study saw value in being able to express themselves in multiple languages, and offered mutual understanding as the most important reason. Group cohesion or identity was another important reason mentioned by participants in the current study, and corresponds to some degree with the theme of social cohesion identified by Coetzee-Van Rooy (2016). Many participants also viewed their ability to express themselves in certain languages as part of their identity, affirming the link between language and identity (Bristowe et al. 2014; McKinney 2013; Ndlangamandla 2010).

Regarding barriers to the use of various languages, these related both to the lack of language-specific options available on aided AAC and also to their own literacy skills. The latter corroborates the findings regarding their self-reported skills in reading and writing – these were generally poorer than spoken language comprehension. For participants from non-English backgrounds, this discrepancy was greater for the home language than for English. There may be various reasons. For some participants, the condition that they were diagnosed with or concomitant conditions such as intellectual disability may have complicated the acquisition or re-acquisition of literacy skills. However, it is often difficult to disentangle the effects of inherent capacity from environmental barriers, such as an impoverished home literacy environment, limited or no access to education, limited expectations by caregivers and teachers and no or poor literacy instruction in school (Human Rights Watch 2015; Sturm & Clendon 2004).

Overall, South Africa does not fare well in literacy education at school level, as revealed by the results of the recent Progress in International Reading and Literacy Study (PIRLS, Howie et al. 2017). Although adult literacy rates are reported to be 94% (Statistics South Africa 2017), such statistics have been criticised as misleading because of the challenge in measuring literacy by level of education (Aitchison & Harley 2006; Pretorius 2013). Most participants had at least a level of secondary school education, yet literacy instruction is not always prioritised for learners with disabilities (Human Rights Watch 2015). More importantly maybe, the language of instruction in most South African schools is English from the fourth grade onwards (Department of Basic Education 2011), thereby favouring English rather than home language literacy skill development. African home languages remain languages that are mainly spoken, rather than written (Coetzee-Van Rooy 2012). Providing their children with English rather than home language literacy instruction has also been reported to be a conscious choice of South African caregivers, in view of limited options of SGDs with TTS synthesis available for other languages (Tönsing et al. 2018; Van Niekerk & Tönsing 2015).

A lack of literacy skills in the home language and other languages that they understand may have significant consequences for persons in need of AAC. For many, literacy constitutes an effective method for linguistic expression, allowing them unrestricted access to express their thoughts and opinions in a way that is understandable to multiple partners, especially if they also have access to an SGD with TTS in the appropriate languages (Light & McNaughton 2013). Those with limited literacy skills may need to rely on unaided methods that typically do not allow linguistic expression, or on their residual speech, which may require a significant amount of guessing and interpretation by partners, and may only be effective when partners are very familiar with the person (Dowden 1997). Both these methods would severely restrict communication access.

Alternatively, aided AAC systems that do not require literacy skills (such as those based on picture symbols) may be used. However, if such systems are required to give access to anything remotely approaching the expressive power of spoken or written language, they typically require AAC- and language-specific expertise to design, individualise and maintain, as a great number of words, concepts and possibly also grammatical markers need to be organised and stored to allow the generation of meaningful novel sentences (Light & McNaughton 2012; Thistle & Wilkinson 2013).

### Practical implications

Improved access to expression in multiple languages for South Africans with severe communication disabilities needs to be addressed on a number of levels. Firstly, it is clear that communication technologies and specifically AAC technologies should be designed in such a way as to allow a person to express themselves in multiple languages. Text prediction in South African languages other than English, for example, may assist those with limited literacy skills (Herold, Alant & Bornman 2008). Text-to-speech synthesis in various South African languages available across multiple operating systems and integrated into specific AAC applications could go a long way in giving many South Africans with severe communication disabilities ‘a voice’ (Schlünz et al. 2017). The ever-increasing availability of portable and specifically mobile technology has put potential AAC solutions into the hands of many who could previously not obtain them (McNaughton & Light 2013), although physical access to these devices may require additional adaptations (Bornman et al. 2016).

However, unless AAC applications are available that are able to truly meet communication needs in various contexts (e.g. talking to the family, friends, neighbours and other community members), their use will remain limited. In this regard, South African multilingual AAC systems that do not require literacy skills need to be developed – both for children who are not yet literate but also for adults who may never have had the opportunity to become fully literate in one or more of the languages they understand.

There is as yet no consensus in the AAC field as to ‘the best’ way of designing such systems, and arguably, the diversity of persons in need of AAC precludes the discovery of one such method. A knowledge of both language structure and also of the person’s unique communication needs and categorisation preferences is needed to select and organise vocabulary for an AAC system (Baker & Chang 2006; Light & Drager 2007). The experience of South African AAC service providers (Tönsing et al. 2018) as well as comparisons between English and isiZulu core vocabulary (most frequently used vocabulary as determined from transcriptions of natural conversations) (Mngomezulu 2017) confirm the limitations of translation of English systems into other languages – especially into those with a different linguistic typology. Continued collaboration between the main stakeholders (i.e. persons in need of AAC and their families), service providers, linguists and human language technology specialists could assist in developing various AAC system templates or user profiles that can then be adjusted and customised for individuals. Studies are then also needed to determine effective ways of implementing such systems in a variety of contexts.

Furthermore, the results once again underline the need to interrogate literacy teaching practices in the South African education system as suggested by Howie et al. (2017). The PIRLS has been criticised for excluding students with disabilities, thereby maintaining ‘the oppression of low expectation’ (Schuelka 2013:216). We would suggest that learners with disabilities be urgently added to the groups of children identified by Howie et al. (2017) as having a high risk for poor literacy outcomes in South Africa. The choices regarding language of instruction (and, by implication, choices about literacy learning in different languages) in the basic education system made by parents and school governance bodies remain challenging and fraught with controversies (De Klerk 2002a, 2002b). We would urge educators, parents and interventionists to consider long-term consequences specifically for learners who require AAC. Literacy learning opportunities for adults who would like to gain literacy skills in one or more languages should also be further investigated, as suggested by Martin and Murray (2011).

The right to communicate using different languages may need to be recognised more formally in AAC practice guidelines. Although the right to communicate is pertinently upheld in the AAC community (e.g. the Communication Bill of Rights, cf. Brady et al. 2016), the rights of persons using AAC to express themselves in languages of their choosing seem not to have received equal attention.

## Limitations

This exploratory study has a number of limitations. The sample was small, and was recruited via an email list and an empowerment programme. The use of an English questionnaire biased the sample of participants towards those with literacy and English language skills, further limiting the generalisability of the results. The sample proportionally over-represented participants from English or Afrikaans language backgrounds. Both resource limitations and the estimated unlikelihood of access to AAC services for persons who were not reached via these recruitment methods precluded us from using more comprehensive sampling methods. The persisting and widening economic divide which continues along racial lines, as well as lack of access to formal education and AAC service delivery in languages other than English (Kathard et al. 2011) still leaves a large proportion of persons with severe communication disabilities underserved, with no access to appropriate intervention.

Multiple methods of collecting the data were used in the study, and these may have had an influence on the results. Qualitatively, it seemed that there were no differences in the number of inconsistencies or missing data when comparing the questionnaires received via fax or electronically to those administered in a face-to-face interview. Because of the fact that the questionnaire targeted multiple manifest variables that were not related to a few underlying latent constructs, statistical comparisons between data received via the various methods were not carried out.

Two participants with a limited understanding of English were included in the study, and the questions were translated into their home language by a trained research assistant and a personal assistant, respectively. The personal assistant worked as a receptionist and assistant at a clinic for children with disabilities and their families based at a provincial hospital and was experienced in English–Setswana translation as part of her work. However, the informal translation procedures used remain a significant limitation.

Social desirability effects are also typically a limitation of survey designs, and may have been exacerbated by the fact that many questionnaires were administered as interviews led by research assistants, who were familiar with the participants through their involvement in the communication empowerment programme. Although the administration of questionnaires with assistance from research assistants and others facilitated access to the study for many participants (e.g. those who would have found writing or typing to complete the whole questionnaire independently too physically tiring), their involvement also poses a risk to fidelity, as they may have added their own interpretations to the participants’ answers. All but five respondents were personally known to the first author, posing a further risk to social desirability effects. The perspectives of the researchers on multilingual issues in AAC were not known to participants, and it was emphasised in the information letter that the participants’ own views were sought, without there being correct or incorrect answers. Participants in the empowerment programme were also assured that the way in which they answered would not in any way influence their future involvement in the programme.

Collecting additional information on the communication skills and physical abilities through formal measures such as the Functional Communication Classification System (Barty, Caynes & Johnston 2016), the Manual Ability Classification System (Eliasson et al. 2006) or the Gross Motor Functional Classification System (Palisano et al. 2008) would have provided a more comprehensive picture of the participants and their abilities.

## Conclusions

The findings from this exploratory study suggest that South African adults using AAC understand multiple spoken languages but face limitations in their ability to express themselves in multiple languages using AAC. These limitations seemed related both to a lack of appropriate AAC systems (e.g. a lack of TTS in African languages other than English), as well as the adults’ limited literacy skills in some of the languages they understood. Most adults desired additional or increased access to expression in various languages. Particularly participants from non-English backgrounds desired access or increased access to their home language. Appropriate multilingual AAC systems and AAC intervention are therefore urgently required. Appropriate literacy learning opportunities for both adults and children who need or use AAC also need to be created, as literacy skills can give access to autonomous communication.

## Footnotes

1 Text-to-speech synthesis entails the conversion of text on an electronic device to computerised speech.

2 Picture communication symbols are a product of Mayer Johnson, part of the Tobii Dynavox family.www.mayer-johnson.com

3 SymbolStix are a product of n2y, www.n2y.com

4 Widgit symbols are a product of Widgit Software, www.widgit.com

5 Minspeak^®^ is a product of Semantic Compaction Systems, www.minspeak.com

## References

[CIT0001] Aitchison J. & Harley A., 2006, ‘South African illiteracy statistics and the case of the magically growing number of literacy and ABET learners’, *Journal of Education* 39(1), 90–112, viewed 16 October 2018, from https://journals.co.za/content/joe/39/1/AJA0259479X_93

[CIT0002] American Speech-Language-Hearing Association, 2018, *Augmentative and alternative communication*, viewed 5 May 2018, from https://www.asha.org/NJC/AAC/

[CIT0003] Baker B.R. & Chang S-K., 2006, ‘A Mandarin language system in augmentative and alternative communication (AAC)’, *International Journal of Computer Processing of Languages* 19(4), 225–237. 10.1142/S0219427906001438

[CIT0004] Barty E., Caynes K. & Johnston L.M., 2016, ‘Development and reliability of the functional communication classification system for children with cerebral palsy’, D*evelopmental Medicine and Child Neurology* 58(10), 1036–1041. 10.1111/dmcn.13124 27087436

[CIT0005] Bordieu P., 1991, *Language and symbolic power*, transl. Raymond G. & Adamson M., Polity Press, Cambridge.

[CIT0006] Bornman J., Bryen D.N., Moolman E. & Morris J., 2016, ‘Use of consumer wireless devices by South Africans with severe communication disability’, *African Journal of Disability* 5(1), 1–9. 10.4102/ajod.v5i1.202 PMC5433450 28730045

[CIT0007] Brady N.C., Bruce S., Goldman A., Erickson K., Mineo B., Ogletree B.T. et al., 2016, ‘Communication services and supports for individuals with severe disabilities: Guidance for assessment and intervention’, *American Journal on Intellectual and Developmental Disabilities* 121(2), 121–138. 10.1352/1944-7558-121.2.121 26914467 PMC4770561

[CIT0008] Bridges S., 2004, ‘Multicultural issues in augmentative and alternative communication and language: Research to practice’, *Topics in Language Disorders* 24(1), 62–75, viewed 16 October 2018, from http://journals.lww.com/topicsinlanguagedisorders/Abstract/2004/01000/Multicultural_Issues_in_Augmentative_and.7.aspx

[CIT0009] Bristowe A., Oostendorp M. & Anthonissen C., 2014, ‘Language and youth identity in a multilingual setting: A multimodal repertoire approach’, *Southern African Linguistics and Applied Language Studies* 32(2), 229–245. 10.2989/16073614.2014.992644

[CIT0010] Coetzee-Van Rooy S., 2012, ‘Flourishing functional multilingualism: Evidence from language repertoires in the Vaal Triangle region’, *International Journal of the Sociology of Language* 2012(218), 87–119. 10.1515/ijsl-2012-0060

[CIT0011] Coetzee-Van Rooy S., 2016, ‘Multilingualism and social cohesion: Insights from South African students (1998, 2010, 2015)’, *International Journal of the Sociology of Language* 2016(242), 239–265. 10.1515/ijsl-2016-0041

[CIT0012] Dada S., Murphy Y. & Tönsing K., 2017, ‘Augmentative and alternative communication practices: A descriptive study of the perceptions of South African speech-language therapists’, *Augmentative and Alternative Communication* 33(4), 189–200. 10.1080/07434618.2017.1375979 28934864

[CIT0013] De Klerk V., 2002a, ‘Language issues in our schools: Whose voice counts? Part 1 : The parents speak’, *Perspectives in Education* 20(1), 1–14.

[CIT0014] De Klerk V., 2002b, ‘Part 2 : The teachers speak’, *Perspectives in Education* 20(1), 15–28.

[CIT0015] De Valenzuela J.S., Kay-Raining Bird E., Parkington K., Mirenda P., Cain K., MacLeod A.A. et al., 2016, ‘Access to opportunities for bilingualism for individuals with developmental disabilities: Key informant interviews’, *Journal of Communication Disorders* 63, 32–46. 10.1016/j.jcomdis.2016.05.005 27814796

[CIT0016] Department of Basic Education, 2011, *The status of the language of learning and teaching (LOLT) in South Afircan public schools*, Department of Basic Education, Pretoria.

[CIT0017] Dowden P., 1997, ‘Augmentative and alternative communication: Decision making for children with severely unintelligible speech’, *Augmentative and Alternative Communication* 13, 48–58. 10.1080/07434619712331277838

[CIT0018] Drysdale H., Van der Meer L. & Kagohara D., 2015, ‘Children with autism spectrum disorder from bilingual families: A systematic review’, *Journal of Autism and Developmental Disorders* 2(1), 26–38. 10.1007/s40489-014-0032-7

[CIT0019] Eliasson A.C., Krumlinde S., Undholm L., Rösblad B., Beckung E., Arner M. et al., 2006, ‘The Manual Ability Classification System (MACS) for children with cerebral palsy: Scale development and evidence of validity and reliability’, *Developmental Medicine and Child Neurology* 48, 549–554. 10.1017/S0012162206001162 16780622

[CIT0020] Grosjean F., 2013, ‘Bilingualism: A short introduction’, in Grosjean F. & Li P. (eds.), *The psycholinguistics of bilingualism*, pp. 1–6, Wiley-Blackwell, Chichester.

[CIT0021] Gutierrez-Clellen V.F., 1999, ‘Viewpoint. Language choice in intervention with bilingual children’, *American Journal of Speech-Language Pathology* 8(4), 291–302. 10.1044/1058-0360.0804.291

[CIT0022] Harrison-Harris O.L., 2007, ‘AAC, literacy and bilingualism’, *The ASHA Leader* 7(20), 4–17. 10.1044/leader.FTR2.07202002.4

[CIT0023] Herold M., Alant E. & Bornman J., 2008, ‘Typing speed, spelling accuracy, and the use of word-prediction’, *South African Journal of Education* 28, 117–134.

[CIT0024] Howie S.J., Combinck C., Roux K., Tshele M., Mokoena G. & McLeaod Palane N., 2017, *PIRLS literacy 2016: South African highlights report*, viewed 8 February 2018, from http://www.up.ac.za/media/shared/164/ZP_Files/pirls-literacy-2016-hl-report-3.zp136320.pdf

[CIT0025] Huer M.B., Parette H.P. & Saenz T.I., 2001, ‘Conversations with Mexican Americans regarding children with disabilities and augmentative and alternative communication’, *Communication Disorders Quarterly* 22(4), 197–206. 10.1177/152574010102200405

[CIT0026] Human Rights Watch, 2015, *Complicit in Exclusion*, viewed 08 September 2017, from https://www.hrw.org/report/2015/08/18/complicit-exclusion/south-africas-failure-guarantee-inclusive-education-children

[CIT0027] Kamwangamalu N.M., 2003, ‘Globalization of English, and language maintenance and shift in South Africa’, *International Journal of the Sociology of Language* 164, 65–81. 10.1515/ijsl.2003.056

[CIT0028] Kaplan R.B., 2015, ‘Multilingualism vs. monolingualism: The view from the USA and its interaction with language issues around the world’, *Current Issues in Language Planning* 16(1–2), 149–162. 10.1080/14664208.2014.947016

[CIT0029] Kathard H., Pascoe M., Ramma L., Jordaan H., Moonsamy S., Wium A-M. et al., 2011, ‘How can speech-language therapists and audiologists enhance language and literacy outcomes in South Africa? (And why we urgently need to)’, *South African Journal of Communication Disorders* 58, 59–71. 10.4102/sajcd.v58i2.27

[CIT0030] Kay-Raining Bird E., Genesee F. & Verhoeven L., 2016, ‘Bilingualism in children with developmental disorders: A narrative review’, *Journal of Communication Disorders* 63(2016), 1–14. 10.1016/j.jcomdis.2016.07.003 27461977

[CIT0031] Khokhlova I., 2015, ‘Lingua Franca English of South Africa’, *Procedia – Social and Behavioral Sciences* 214, 983–991. 10.1016/j.sbspro.2015.11.689

[CIT0032] Kohnert K., 2013, *Language disorders in bilingual children and adults*, 2nd edn., Plural Publishing, San Diego, CA.

[CIT0033] Kohnert K. & Medina A., 2009, ‘Bilingual children and communication disorders: A 30-year research retrospective’, *Seminars in Speech and Language* 1(212), 219–233. 10.1055/s-0029-1241721 19851950

[CIT0034] Kulkarni S.S. & Parmar J., 2017, ‘Culturally and linguistically diverse student and family perspectives of AAC’, *Augmentative and Alternative Communication* 33(3), 170–180. 10.1080/07434618.2017.1346706 28697629

[CIT0035] Lee J.S. & Wright W.E., 2014, ‘The rediscovery of heritage and community language education in the United States’, *Review of Research in Education* 38, 137–165. 10.3102/0091732X13507546

[CIT0036] Levey S. & Sola J., 2013, ‘Speech-language pathology students’ awareness of language differences versus language disorders’, *Contemporary Issues in Communication Science & Disorders* 40, 8–14.

[CIT0037] Light J.C. & McNaughton D., 2012, ‘Supporting the communication, language and literacy development of children with complex communication needs: State of the science and future research priorities’, *Assistive Technology* 24, 34–44. 10.1080/10400435.2011.648717 22590798

[CIT0038] Light J.C. & McNaughton D., 2013, ‘Literacy intervention for individuals with complex communication needs’, in Beukelman D.R. & Mirenda P. (eds.), *Augmentative and alternative communication: Supporting children and adults with complex communication needs*, 4th edn., pp. 309–351, Paul H Brookes, Baltimore, MD.

[CIT0039] Light J.C. & Drager K., 2007, ‘AAC technologies for young children with complex communication needs: State of the science and future research directions’, *Augmentative and Alternative Communication* 23(3), 204–16. 10.1080/07434610701553635 17701740

[CIT0040] Lozanso-Alonso A., 2017, ‘A cross-generational conversation about the future of teaching Spanish’, *Hispania* 100(5), 213–219. 10.1353/hpn.2018.0052

[CIT0041] Martin A. & Murray J., 2011, ‘It’s never too late to learn: Picture-think’, *Communciation Matters* 25(1), 5–9. 10.1016/S1471-0846(04)00080-0

[CIT0042] May S., 2001, *Language and minority rights*, Pearson Education, Essex.

[CIT0043] McKinney C., 2013, ‘Orientations to English in post-apartheid schooling’, *English Today* 29(1), 22–27. 10.1017/S0266078412000491

[CIT0044] McNaughton D. & Light J.C., 2013, ‘The iPad and mobile technology revolution: Benefits and challenges for individuals who require augmentative and alternative communication’, *Augmentative and Alternative Communication* 29(2), 107–116. 10.3109/07434618.2013.784930 23705813

[CIT0045] Mngomezulu J., 2017, ‘Determining an AAC core vocabulary for Zulu-speaking preschool children’, MA (AAC) thesis, University of Petoria, Pretoria, viewed 16 October 2018, from http://hdl.handle.net/2263/64811.

[CIT0046] Ndlangamandla S.C., 2010, ‘Multilingualism in desegregated schools: Learners’ use of and views towards African languages’, *Southern African Linguistics and Applied Language Studies* 28(1), 61–73. 10.2989/16073614.2010.488444

[CIT0047] Norton B. & Columbia B., 2011, ‘Identity, language learning, and social change’, *Language Teaching* 44(4), 412–446. 10.1017/S0261444811000309

[CIT0048] Palisano R.J., Rosenbaum P., Bartlett D. & Livingston M.H., 2008, ‘Content validity of the expanded and revised Gross Motor Function Classification System’, *Developmental Medicine and Child Neurology* 50(10), 744–750. 10.1111/j.1469-8749.2008.03089.x 18834387

[CIT0049] Pickl G., 2011, ‘Communication intervention in children with severe disabilities and multilingual backgrounds: Perceptions of pedagogues and parents’, *Augmentative and Alternative Communication* 27(4), 229–244. 10.3109/07434618.2011.630021 22136362

[CIT0050] Pretorius S., 2013, ‘SA’s real level of literacy’, *The Citizen*, 29 August, viewed 07 January 2018, from https://citizen.co.za/news/south-africa/31407/literatez/.

[CIT0051] Schlünz G.I., Gumede T., Wilken I., Van Der Walt W., Moors C., Calteaux K. et al 2017, ‘Applications in accessibility of text-to-speech synthesis for South African languages: Initial system integration and user engagement’, in Blignaut P. & Stott T. (eds.), South African Institute of Computer Scientists and Information Technologists (SAICSIT) Proceedings, Bloemfontein, South Africa, September 26–28, 2017, pp. 293–302.

[CIT0052] Schuelka M.J., 2013, ‘Excluding students with disabilities from the culture of achievement: The case of the TIMSS, PIRLS, and PISA’, *Journal of Education Policy* 28(2), 216–230. 10.1080/02680939.2012.708789

[CIT0053] Singh S., Hussein N., Kamal R.M. & Hassan F.H., 2017, ‘Reflections of Malaysian parents of children with developmental disabilities on their experiences with AAC’, *Augmentative and Alternative Communication* 33(2), 110–120. 10.1080/07434618.2017.1309457 28387140

[CIT0054] Soto G. & Yu B., 2014, ‘Considerations for the provision of services to bilingual children who use augmentative and alternative communication’, *Augmentative and Alternative Communication* 30(1), 83–92. 10.3109/07434618.2013.878751 24471987

[CIT0055] Statistics South Africa, 2012, *Census in brief*, Statistics South Africa, Pretoria.

[CIT0056] Statistics South Africa, 2017, *General household survey 2016*, viewed 07 February 2018, from www.statssa.gov.za

[CIT0057] Stewart C.A., 2017, ‘Bilingual AAC intervention: A case study’, Master’s thesis, Department of Speech, Language and Hearing Sciences, University of Colorado.

[CIT0058] Sturm J.M. & Clendon S.A., 2004, ‘Augmentative and alternative communication, language, and literacy: Fostering the relationship’, *Topics in Language Disorders* 24(1), 76–91. 10.1097/00011363-200401000-00008

[CIT0059] The Constitution of the Republic of South Africa, 1996, *Act 108 of 1996*, viewed 08 September 2017, from http://www.justice.gov.za/legislation/constitution/SAConstitution-web-eng.pdf

[CIT0060] Thistle J.J. & Wilkinson K.M., 2013, ‘Working memory demands of aided augmentative and alternative communication for individuals with developmental disabilities’, *Augmentative and Alternative Communication* 29(3), 235–245. 10.3109/07434618.2013.815800 23902430

[CIT0061] Tönsing K.M., Schlünz G., Van Niekerk K. & Wilken I., 2018, ‘AAC services for multilingual populations in SA Service provider perspectives’, *Journal of Communication Disorders* 73, 62–76. 10.1016/j.jcomdis.2018.04.002 29702365

[CIT0062] Van Niekerk K. & Tönsing K., 2015, ‘Eye gaze technology: A South African perspective’, *Disability and Rehabilitation: Assistive Technology* 10(4), 340–346. 10.3109/17483107.2014.974222 25342494

[CIT0063] Webb V., 2010, ‘Multilingualism from below. Really? In South Africa?’, in Cuvellier P., Du PLessis T., Meeuwis M., Vandekerckhove R. & Webb V. (eds.), *Mulilingualism from below*, pp. 134–146, Van Schaik, Pretoria.

[CIT0064] Webb V., Lafon M. & Pare P., 2010, ‘Bantu languages in education in South Africa: An overview. Ongekho akekho! – The absentee owner’, *Language Learning Journal* 38(3), 273–292. 10.1080/09571730903208389

[CIT0065] Yu B., 2013, ‘Issues in bilingualism and heritage language maintenance: Perspectives of minority-language mothers of children with autism spectrum disorders’, *American Journal of Speech-Language Pathology* 22, 10–24. 10.1044/1058-0360(2012/10-0078)a 23071196

